# Enhanced diagnostic accuracy for neurocognitive disorders: a revised cut-off approach for the Montreal Cognitive Assessment

**DOI:** 10.1186/s13195-020-00603-8

**Published:** 2020-04-07

**Authors:** Alessandra E. Thomann, Manfred Berres, Nicolai Goettel, Luzius A. Steiner, Andreas U. Monsch

**Affiliations:** 1 Memory Clinic, University Department of Geriatric Medicine FELIX PLATTER, Burgfelderstrasse 101, CH-4055 Basel, Switzerland; 2 Anesthesiology, University Hospital Basel, Basel, Switzerland; 3 Department of Mathematics and Technology, University of Applied Sciences Koblenz, Koblenz, Germany; 4 Department of Clinical Research, University of Basel, Basel, Switzerland

**Keywords:** Sensitivity and specificity, Neuropsychology, Mental status and dementia tests, Montreal Cognitive Assessment, Mini Mental State Examination, Neurocognitive disorders, ROC curve, Cognitive dysfunction, Area under curve

## Abstract

**Background:**

The Montreal Cognitive Assessment (MoCA) has good sensitivity for mild cognitive impairment, but specificity is low when the original cut-off (25/26) is used. We aim to revise the cut-off on the German MoCA for its use in clinical routine.

**Methods:**

Data were analyzed from 496 Memory Clinic outpatients (447 individuals with a neurocognitive disorder; 49 with cognitive normal findings) and from 283 normal controls. Cut-offs were identified based on (a) Youden’s index and (b) the 10th percentile of the control group.

**Results:**

A cut-off of 23/24 on the MoCA had better correct classification rates than the MMSE and the original MoCA cut-off. Compared to the original MoCA cut-off, the cut-off of 23/24 points had higher specificity (92% vs 63%), but lower sensitivity (65% vs 86%). Introducing two separate cut-offs increased diagnostic accuracies with 92% specificity (23/24 points) and 91% sensitivity (26/27 points). Scores between these two cut-offs require further examinations.

**Conclusions:**

Using two separate cut-offs for the MoCA combined with scores in an indecisive area enhances the accuracy of cognitive screening.

## Background

A steep increase in the prevalence of dementia is expected [1], associated with social, economic, and societal challenges. Early detection of dementia is crucial for an implementation of therapeutic strategies in the earliest disease stages [2], and reliable cognitive screening tools play an important role in this process of identifying individuals with cognitive impairment [3]. In the context of clinical research, accurate cognitive assessment tools are needed for an adequate selection of participants, since erroneous inclusion or exclusion of individuals may bias study findings [4, 5].

The Montreal Cognitive Assessment (MoCA) [6] has gained popularity for cognitive screening. It correlates well with extensive neuropsychological test batteries [7, 8] and covers most of the cognitive domains outlined in the Diagnostic and Statistical Manual, 5th Edition (DSM-5) [9]. However, while the initially proposed cut-off (25/26 points) [6] has shown good sensitivity for mild cognitive impairment (MCI) (i.e., ≥ 83%) [6, 10, 11], this cut-off was found to have a specificity of 66% or less in various different studies, implying a potentially unacceptably high number of false-positive classifications [7, 10–13]. Consequently, new cut-offs have been proposed for various patient populations and languages (see [13] for an overview). However, the psychometric properties of any screening test are not fixed characteristics, but depend on the clinical context [14], limiting the transferability of these cut-offs to other settings. Moreover, most previous authors defined “optimal cut-offs,” which aim at finding the best balance between sensitivity and specificity, as opposed to conventional cut-offs that are based on clinical standards (e.g., test performance 1–2 SD below the normative mean [9, 15]). Optimal cut-offs are likely to be sample-dependent and specific to the individual study [15, 16] and should therefore be validated in independent samples. Furthermore, in most validation studies, a rather homogenous patient sample was recruited (e.g., only patients with probable Alzheimer’s disease (AD) according to McKhann criteria [17], exclusion of patients with medical comorbidities), which does not reflect the clinical reality, where patient populations are typically heterogeneous, and medical comorbidities are frequent. In addition, excluding patients who are difficult to diagnose induces several forms of bias and may lead to an overestimation of diagnostic accuracy [18, 19]. Heterogeneous samples reflect the clinical reality more accurately as healthcare professionals face the challenge to identify truly impaired patients from a pool of individuals with a suspected neurocognitive disorder (NCD), irrespective of the underlying cause.

In the present study, we aim to address these limitations and therefore estimate the diagnostic accuracy of the original MoCA cut-off in a sample of consecutively referred Memory Clinic outpatients (MC sample). The objective of the study was to differentiate normal findings (NF; i.e., neurocognitive results were within normal limits) from patients with mild and major NCD (labeled Mild+Major NCD in the following) [9]. Since the MoCA was developed to identify individuals with MCI, subgroup analyses are performed for patients diagnosed with mild NCD (labeled Mild NCD in the following). Given the high rate of false-positive classifications that is associated with the original MoCA cut-off, we aimed at finding a new cut-off with higher specificity. In this context, we introduce an approach to determine a conventional cut-off solely based on a sample of cognitively healthy normal controls (NCs), which we then validated in the MC sample. We then compare this conventional cut-off to an optimal cut-off approach.

In sub-analyses, we investigate the differences in diagnostic accuracy in relation to demographic adjustments by comparing the original MoCA score with recently established demographically corrected MoCA *z*-scores [7] (an Excel file for the calculation of the *z*-score is provided in the Supplementary material). Finally, during our analyses, we noticed that information is lost when a continuous variable like the MoCA is dichotomized [20, 21], and a traditional binary cut-off is used. We therefore propose a revised approach to evaluate cognitive performance on the MoCA using two separate cut-offs in combination with an indecisive area between these scores.

## Methods

### Participants

We retrospectively assessed data from 1307 consecutive outpatients of the Memory Clinic, University Department of Geriatric Medicine FELIX PLATTER, Basel, Switzerland, undergoing neuropsychological assessment between March 6, 2017, and October 12, 2018. Data from patients meeting the following inclusion criteria were considered for the analysis: (a) age ≥ 65 years, (b) education ≥ 7 years, (c) fluency in the German language, and (d) availability of a neuropsychological assessment including the Mini Mental Status Examination (MMSE) [22] and the German version of the MoCA. Exclusion criteria were (a) severe sensory or motor impairment interfering with cognitive testing, (b) repeated testing with the MoCA due to follow-up examinations, and (c) documented refusal of the use of personal health-related data for research purposes. An overview of the clinical diagnoses is provided in Supplementary Table 1. The demographic inclusion criteria were selected to match the NC group from a previous normative study on the German version of the MoCA (see [7] for details).

The NC group was recruited from an existing Registry of Individuals Interested to Participate in Research established by the Memory Clinic in 2013 with approval from the local ethics committee (no. EKBB 280/12). From this registry, potential participants with the required demographic characteristics (age, education, sex) were identified and invited to participate in a normative study on the German verison of the MoCA [7]. During the recruitment process, a stratification of sex (female and male) and age (groups: 65–69, 70–74, 75–79, and > 79 years) was applied. Inclusion criteria were (a) age ≥ 65 years, (b) education ≥ 7 years, (c) fluent German speaking, and (d) provided written informed consent. Subjects who met one of the following criteria were excluded: (a) cognitive impairment (i.e., MMSE < 27/30 and/or Consortium to Establish a Registry for Alzheimer’s Disease-Neuropsychological Assessment Battery (CERAD-NAB) < 85.89 [23], any diagnosis of cognitive impairment), (b) diagnosis and/or symptoms of depression (i.e., Geriatric Depression Scale (GDS) > 5/15 [24]), (c) severe sensory or motor impairment interfering with cognitive testing, (d) serious somatic disease, (e) any disease or events affecting the central nervous system, (f) cerebrovascular disease, (g) current medication with psychoactive drugs except for benzodiazepines, and (h) participation in a cognitive study within the last 3 months (to avoid practice effects).

### Procedures

Patients were assessed in the following order: (a) detailed patient and medical history, (b) neuropsychological screening including the MMSE and the clock drawing test, (c) German version of the MoCA, (d) assessment of symptoms of depression (15-item GDS or Beck Depression Inventory) [25], and (e) extensive neuropsychological examination. Neuropsychological assessments were performed by board-certified neuropsychologists and by psychologists with a master’s degree in psychology. Neuropsychological test results were interpreted based on demographically corrected (i.e., age, sex, and education) *z*-scores and were used to inform diagnostic deliberations. The patients were medically examined by a neurologist or a geriatrician. Imaging (i.e., structural magnetic resonance imaging, computed tomography, and/or positron emission tomography with ^18^F-fluorodeoxyglucose) was performed, and in some patients, cerebrospinal fluid was collected to assess for protein deposition. Diagnostic consensus was reached in weekly interdisciplinary meetings by neuropsychologists, neurologists, neuroradiologists, nuclear medicine specialists, geriatricians, psychiatrists, and a neuropathologist. MoCA results were not considered in the diagnostic process.

The detailed procedures for the NC group are described elsewhere [7]. Briefly, study eligibility in the NC group was assessed by the German versions of the MMSE and the 15-item GDS questionnaire. All individuals were then assessed with the German version of the MoCA, followed by the German version of the CERAD-NAB. Subjects meeting any exclusion criteria were omitted from the main analysis.

The study protocol (no. EKNZ 2018-00737) was approved by the regional research ethics board (*Ethikkommission Nordwest- und Zentralschweiz* [EKNZ]) on May 22, 2018. The study was conducted in respect of the most recent version of the Declaration of Helsinki and was registered on ClinicalTrials.gov (NCT03581643). The need for informed consent was waived by the EKNZ.

### Statistical analyses

Demographical characteristics and test scores were compared pairwise using the non-parametric Wilcoxon rank sum test for between-group comparisons. Differences in sex were analyzed using the chi-squared test. All statistical analyses were performed using R, version 3.5.0 (R Foundation, Vienna, Austria) and RStudio Desktop (RStudio, Boston, MA, USA). Data are presented as mean (SD), and the education-corrected MoCA score (+ 1 point for < 12 years of education) was used, unless stated otherwise. There were no missing data in any of the analyses.

#### Different cut-off approaches for the MoCA

Diagnostic accuracies were calculated in the MC sample (i.e., Mild+Major NCD vs. NF, and Mild NCD vs. NF), for cut-offs on (a) the MoCA score and (b) the MoCA *z*-score. The different approaches to calculate the cut-offs are outlined in the following sections: “Original MoCA cut-off,” “Balanced cut-off,” “Youden’s index,” and “10th percentile in NCs.” For the MMSE, cut-offs were calculated in the MC sample (again Mild+Major NCD vs. NF, and Mild NCD vs. NF), using Youden’s index (see the section “Youden’s index”).

##### Original MoCA cut-off

The original MoCA cut-off (25/26 points) proposed by Nasreddine et al. [6] was applied to the MC sample, and diagnostic accuracies were calculated for (a) Mild+Major NCD vs. NF and (b) Mild NCD vs. NF.

##### Balanced cut-off

A balanced cut-off was calculated by choosing the scores where values of sensitivity and specificity are as equal as possible. Again, this approach was applied to the MC sample and diagnostic accuracies were derived for the MoCA score and the MoCA *z*-score for (a) Mild+Major NCD vs. NF and (b) Mild NCD vs. NF.

##### Youden’s index

Using the Optimal Cutpoints Package in R [26], Youden’s index (sensitivity + specificity − 1) [27] was applied to define the optimal cut-offs in the MC sample (Mild+Major NCD vs. NF and Mild NCD vs. NF) for the MoCA score, the MoCA *z*-score, and the MMSE. Youden’s index is calculated for every potential cut-point on the MoCA/MMSE; the cut-off where Youden’s index reaches its maximum value (i.e., the highest possible Youden’s index would be sensitivity = 100 + specificity = 100 – 1 = 199) is selected as the optimal cut-off. This approach considers false-positive classifications as undesirable as false-negative ones and aims at finding the cut-off with the overall highest sensitivity and specificity [27].

##### 10th percentile in NCs

For this approach, MoCA cut-offs were derived solely based on the NC group. The cut-off was selected by pre-defining a desired specificity of approximately 90% in the NC group (i.e., maximum 10% false-positive classifications in a group of cognitively healthy individuals). This was achieved by choosing the MoCA score and the MoCA *z*-score that split the NC sample at the 10th percentile. In this approach, normality is defined as a reference range based on the distribution of scores in cognitively healthy individuals, and scores below the 10th percentile were considered pathological. The resulting cut-offs were then validated in the MC sample to differentiate Mild+Major NCD vs. NF and Mild NCD vs. NF.

#### Determination of the overall diagnostic accuracy

The discriminative power of the MoCA score, the MoCA *z*-score, and the MMSE score was estimated in terms of area under the curve (AUC) in the MC sample. Receiver operating characteristic (ROC) curves were calculated using the pROC package in R [28]. The AUCs of the MoCA score vs. MoCA *z*-score, the MoCA score vs. MMSE score, and the MoCA *z*-score vs. MMSE score were compared with a bootstrap two-sided significance test for correlated ROC curves. The correct classification rates of the newly derived MoCA cut-offs were compared to the original MoCA cut-off and the optimal cut-offs on the MMSE using McNemar’s test. Results were corrected for multiple comparisons according to Bonferroni-Holm.

#### Definition of two cut-offs and an indecisive area for the MoCA

During our analyses, we realized that the presented approaches to calculate cut-offs were not satisfying, since either specificity, sensitivity, or both were low. Therefore we considered to introduce a multiple cut-off approach. First, we created a plot to visualize the relationship between MoCA scores and rates of sensitivity and specificity to better understand which cut-offs should be selected. We aimed at finding cut-offs with approximately 90% sensitivity and 90% specificity. This plot is thereafter referred to as “Two cut-offs and an indecisive area.” For this purpose, cumulative frequencies were calculated separately for Mild NCD and for NC for each MoCA score. Thus, the proportion of individuals who performed equally or below a given score was determined for each score and expressed in percent of the whole sample. The cumulative frequency for a given score in Mild NCD corresponds to the sensitivity. Specificity is represented by the complementary sum (1 − cumulative frequency) in NC.

## Results

### Descriptive analysis

Four hundred and forty-seven patients (Mild+Major NCD), 49 normal findings (NF), and 283 normal controls (NC) were included in the final analysis. Demographic characteristics are displayed in Table 1. There were no differences between NC and NF. Compared to NF, the patients (i.e., Mild+Major NCD, Mild NCD) were older (*P* value < .001), had fewer years of formal education (*P* value < .001), and had lower test scores (MMSE: *P* value < .001; MoCA: *P* value < .001, MoCA *z*-score: *P* value < .001). There were no sex differences between the groups.

**Table 1:** Demographic characteristics

Group	NC	NF	Mild+Major NCD	Mild NCD
***n***	283	49	447	159
**Prevalence in MC sample %**	–	9.9	90.1	32.1
**Age mean (SD)**	73.8 (5.2)	73.1 (5.6)	78.3 (5.9)*	76.0 (6.0)*
**Age range**	65–91	65–88	65–91	65–91
**Education mean (SD)**	13.6 (2.9)	13.8 (2.7)	12.2 (3.0)*	12.4 (3.1)*
**Education**	7–20	8–20	7–20	7–20
**Female %**	54.8	40.8	55.7	53.5
**MMSE score**	29.2 (0.9)	29.0 (1.0)	25.1 (3.5)*	27.2 (2.2)*
**MoCA score**	26.5 (2.4)	26.5 (2.2)	19.1 (4.5)*	22.0 (3.6)*
**MoCA score range**	16–30	22–30	2–30	12–30
**MoCA*z*-score**	0.0 (1.0)	0.1 (1.0)	− 2.1 (1.0)*	− 1.5 (1.0)*
**MoCA*z*-score range**	− 3.0–2.4	− 1.7–1.9	− 4.3–1.5	− 3.7–1.5

Data are presented as mean (SD). There were no differences between NC and NF. Mild NCD is a subgroup of Mild+Major NCD. NF is compared to Mild+Major NCD and Mild NCD: *P < .001

*Abbreviations*: *NC* normal controls, *NCD* neurocognitive disorder, *NF* normal findings, *MMSE* Mini Mental State Examination, *MoCA* Montreal Cognitive Assessment; *MoCA z-score* demographically corrected standard score [7]

### Diagnostic accuracies

ROC curves for the MC sample are displayed in Supplementary Fig. 1. The AUC of the MoCA scores appears larger than that of the MMSE. However, with the application of the Bonferroni-Holm procedure, the AUC neither differed significantly between MoCA and MMSE scores (MoCA [AUC = 0.94] vs. MMSE [AUC = 0.84]: *P* value = .051; MoCA *z*-score [AUC = 0.94] vs. MMSE: *P* value = .074) nor between the uncorrected MoCA and the MoCA *z*-score (*P* value = 1.0).

Cut-offs and the corresponding diagnostic properties for the MoCA score, the MoCA *z*-score, and the MMSE are provided in Table 2. A MoCA score of 23/24 points was the optimal cut-off according to the 10th percentile method as well as according to Youden’s index in all patient groups. This cut-off had better correct classification rates than the original MoCA cut-off (25/26 points; *P* value < .001) and the MMSE score (*P* value < .001) in both patient samples. Specificity for the cut-off of 23/24 points was high with 92%, and it had good sensitivity for Mild+Major NCD (84%). However, sensitivity was low for Mild NCD (65%). The original MoCA cut-off (25/26 points) had high sensitivity for Mild+Major NCD (94%) and for Mild NCD (86%), but poor specificity (63%). For Mild NCD, an intermediate (i.e., balanced) cut-off (24/25 points) had neither good sensitivity (74%) nor good specificity (74%). We, therefore, aimed at obviating this trade-off between sensitivity and specificity by defining two separate cut-offs. This is illustrated by the example of Mild NCD vs. NC in the “Two separate cut-offs and an indecisive area” section.

**Table 2:** Cut-offs and diagnostic accuracy for the MoCA score, the MoCA *z*-score, and the MMSE

Group	Mild+Major NCD vs. NF	Mild NCD vs. NF
**MoCA score**		
**AUC (95% CI)**	**0.94 (0.91–0.96)**	**0.86 (0.81–0.91)**
**Original cut-off**	**25/26**	**25/26**
Correct classification rate^†^	79%	75%
Sensitivity (95% CI)	94% (94–95%)	86% (84–87%)
Specificity (95% CI)	63% (60–67%)	63% (60–67%)
**Balanced cut-off**	**24/25**	**24/25**
Correct classification rate^†^	82%	74%
Sensitivity (95% CI)	90% (89–90%)	74% (72–76%)
Specificity (95% CI)	74% (70–77%)	74% (70–76%)
**Cut-off; Youden’s index**^‡^	**23/24**	**23/24**
**Cut-off; 10th percentile in NCs**	**23/24**	**23/24**
Correct classification rate^†^	88%	79%
Sensitivity (95% CI)	84% (83–85%)	65% (63–67%)
Specificity (95% CI)	92% (90–94%)	92% (90–94%)
MoCA*z*-score*		
**AUC (95% CI)**	**0.94 (0.91–0.96)**	**0.86 (0.81–0.91)**
**Balanced cut-off**	**≤ − 1.08**	**≤ − 0.84**
Correct classification rate^†^	86%	76%
Sensitivity (95% CI)	86% (85–87%)	76% (74–78%)
Specificity (95% CI)	86% (83–89%)	76% (73–79%)
**Cut-off; Youden’s index**^‡^	**≤ − 1.31**	**≤ − 1.14**
Correct classification rate^†^	**88%**	**79%**
Sensitivity (95% CI)	81% (80–82%)	68% (66–70%)
Specificity (95% CI)	94% (92–96%)	90% (88–92%)
**Cut-off; 10th percentile in NCs**	**≤ − 1.36**	**≤ − 1.36**
Correct classification rate^†^	**88%**	**79%**
Sensitivity (95% CI)	80% (79–81%)	61% (59–63%)
Specificity (95% CI)	96% (95–97%)	96% (95–97%)
**MMSE**		
**AUC (95% CI)**	**0.89 (0.85–0.93)**	**0.78 (0.72–0.85)**
**Cut-off; Youden’s index**^‡^	**27/28**	**28/29**
Correct classification rate^†^	82%	73%
Sensitivity (95% CI)	72% (71–73%)	69% (67–70%)
Specificity (95% CI)	92% (90–94%)	76% (72–79%)

*Abbreviations*: AUC area under the curve, *CI* confidence interval, *NC* normal controls, *NCD* neurocognitive disorder, *NF* normal findings

^†^Correct classification rate = (sensitivity + specificity)/2

^‡^Youden’s index = sensitivity + specificity − 1

*MoCA *z*-score = demographically corrected standard score [7]

### Two separate cut-offs and an indecisive area

**Fig. 1:**
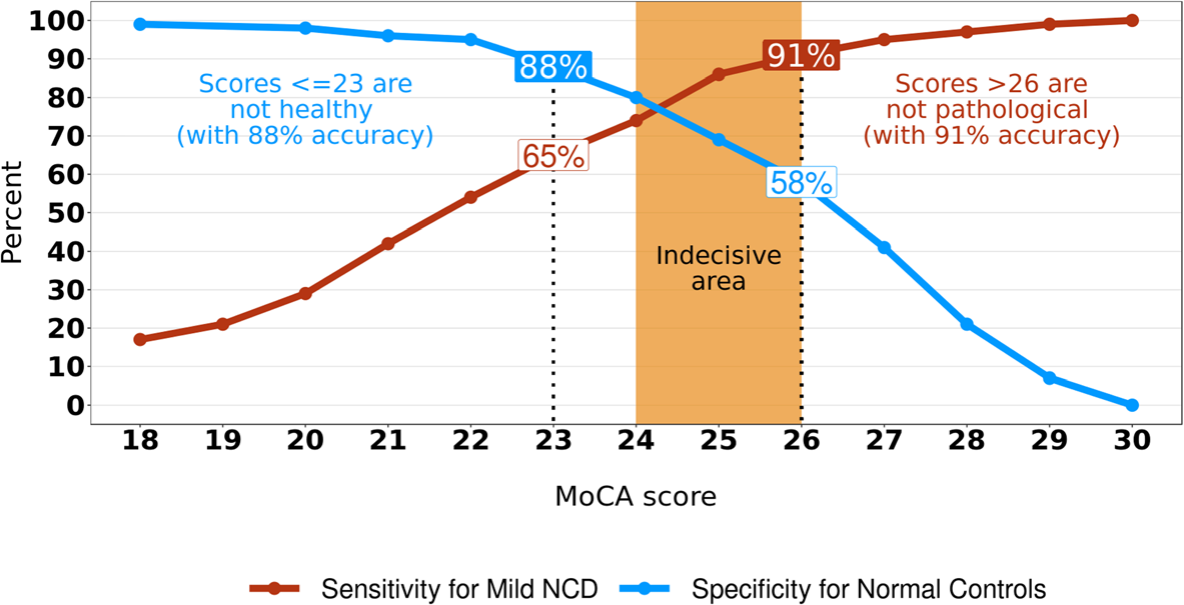
Two separate cut-offs and an indecisive area. The percentage of patients with Mild NCD who were correctly classified as patients (sensitivity, red line) and the percentage of normal controls that were correctly classified as normal controls (specificity, blue line) are illustrated. Two cut-offs are highlighted by the dashed lines: one cut-off for not-healthy results (23/24; with 88% specificity) and one cut-off for not-pathological results (26/27; with 91% sensitivity). Scores between these two cut-offs constitute an indecisive area (in orange), where information from further examinations is required. The + 1 adjustment for individuals with education < 12 years proposed by Nasreddine et al. was applied to calculate the MoCA score [6]

**Fig. 2:**
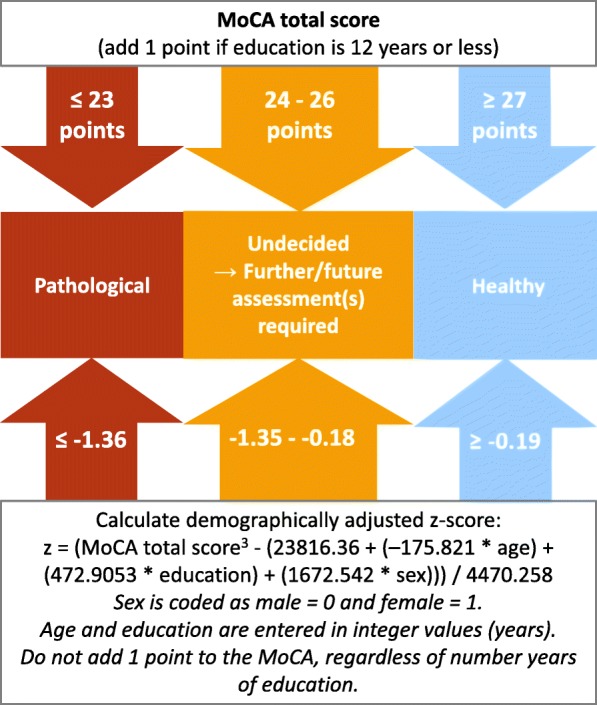
Decision tree for clinical evaluations of the MoCA score and the MoCA *z*-score. This decision tree may be used to determine which cut-offs for the MoCA score and the MoCA *z*-score are most appropriate. The cut-offs proposed here were based on the classification of normal controls (NC) vs. mild neurocognitive disorder (Mild NCD)

In Fig. 1, sensitivity based on Mild NCD is plotted against specificity based on NC. Specificity increases with lower scores, while sensitivity increases with higher scores. At 23/24 points, specificity is 88%, indicating that only 12% of the NC scored ≤ 23 points. At 26/27 points, sensitivity is 91%, so only 9% of patients with Mild NCD achieved scores > 26 points. Consequently, pathological and normal cognition may be defined using two separate cut-offs. Analogous to the concept of *z*-scores, a distribution of scores is assumed, and extreme values are considered improbable for a specific population. Among those who are cognitively healthy, values below a given cut-off (i.e., 23 points) are rare, suggesting that an individual scoring ≤ 23 points is probably not healthy. This statement was accurate in 88% of the NC group (= specificity). Values above a given cut-off (i.e., > 26 points) are uncommon in Mild NCD. Therefore, an individual who attains > 26 points on the MoCA probably does not suffer from an NCD. This statement was accurate in 91% of Mild NCD patients (= sensitivity). Scores between these two cut-offs (24, 25, and 26 points) constitute an indecisive area. This indecisive area may be greater or smaller, depending on the desired accuracies (i.e., for sensitivity of 95% and specificity of 95%, the indecisive area would encompass MoCA scores from 23 to 26 points). In Fig. 2, we illustrate these two cut-offs together with an indecisive area to provide clinicians a means to determine which cut-offs are most appropriate with their patients. The corresponding positive predictive values (PPV) and negative predictive values (NPV) are plotted in Supplementary Fig. 2.

## Discussion

The German MoCA showed good AUC, sensitivity, and specificity for the classification of patients with mild and major NCD versus cognitively healthy normal findings when applied in a heterogeneous group of individuals referred to a university-affiliated Memory Clinic. In the present study, a MoCA score of 23/24 points was established as the optimal cut-off across different patient groups based on two methods. This finding is in line with a recent meta-analysis including seven validation studies on the MoCA [13]. The new MoCA cut-off had an improved correct classification rate compared to both, the original MoCA cut-off and the MMSE. Further, differences in diagnostic accuracy depending on the severity of cognitive impairment (Mild vs. Major NCD) were revealed. While a cut-off of 23/24 points had high sensitivity for all patients (Mild+Major NCD [84%]), sensitivity was low for Mild NCD (65%). When applying a higher cut-off (e.g., the originally proposed 25/26 points), sensitivity for Mild NCD increased to 91%; however, specificity to detect NF was low (59%). If both measures are balanced, neither of them is sufficiently high. Indeed, most screening tools for MCI lack either sensitivity or specificity [29].

In the current situation, it is difficult to favor one above the other. Specificity should be high to avoid falsely classifying healthy individuals as cognitively impaired and potentially provoke distress and insecurities in the affected individuals and their families. This is especially important in the context of AD and other neurodegenerative diseases, which cannot be reversed at this point of time. However, one may also argue in favor of prioritizing sensitivity, since some of the underlying etiologies of mild NCD (e.g., sleep apnea) may be easily treated and potentially even reversed. Moreover, in the context of mild NCD—regardless of the underlying etiology―various options to compensate for the presence of mild NCD exists and early diagnosis of cognitive decline may improve an individuals and his/her relatives’ quality of life. Nevertheless, ideally sensitivity and specificity should be balanced. Based on the findings of this study, we therefore propose a revised method to evaluate cognitive performance, taking the MoCA as an example. This builds up on previous efforts of other authors having developed demographic adjustments for the MoCA as a reaction of the too stringent nature of the original MoCA cut-off (e.g., [30, 31], and [7] for an overview of previously published normative studies). Instead of applying a single cut-off, two separate cut-offs may be used. One cut-off for results that are unlikely within the normal range and one cut-off for scores that are rarely seen in patients. MoCA scores > 26 points may be considered as not pathological with very high accuracy, while scores ≤ 23 points are very likely not healthy. Between these scores, we have defined an indecisive area. When an individual scores between 24 and 26 points (i.e., within the indecisive area), the clinician should start a more comprehensive neuropsychological assessment (e.g., an in-depth assessment according to DSM-5) [9] or reassess the individual with the MoCA in approximately 6 to 12 months.

### Choice of normative samples and patient characteristics

It has been argued that a restrictive cognitively healthy normative group may not be entirely comparable to the population, which is typically screened with the MoCA. This may artificially boost specificity of a test and lead to an overestimation in diagnostic accuracy [16, 19]. We addressed this issue by analyzing two groups of cognitively healthy individuals: one that was purposely recruited for a previous normative study (NC) and one that was formed by consecutively referred patients with a cognitive normal finding (NF). In our study, there were no differences between the NC and the NF group, neither in demographic characteristics nor in cognitive performance. Furthermore, the optimal MoCA cut-offs were identical in these two groups. This suggests that the healthy controls in our study are representative for individuals with cognitive normal findings in the clinical routine. While this is reassuring, longitudinal data from individuals, who remained healthy for several years, should be analyzed in future studies.

### Influence of demographic adjustments on diagnostic accuracy

In our study, individuals with mild and major NCD were older and had less years of formal education when compared to the NF group. We deliberately chose not to match the groups on demographic characteristics to reflect the true nature of this clinical routine sample. Moreover, while some authors (including our group) have suggested that correcting for demographical effects may increase diagnostic accuracy when evaluating cognitive performance [7, 13], others have questioned the utility of demographical adjustments [32], since age and education are per se risk factors of cognitive decline. In the current study, we addressed this subject by comparing demographically adjusted MoCA *z*-scores (accounting for age, education, and sex) with the MoCA raw total score (without any demographic corrections). Conversely, we found no difference between demographically corrected and uncorrected MoCA scores in the overall diagnostic accuracy measured by the AUC. However, a difference emerged in the balance of sensitivity and specificity. When considering the effects of age, education, and sex (*z*-scores), the MoCA gained specificity, while the uncorrected MoCA score showed increased sensitivity. The education-corrected MoCA score was located in between, with higher sensitivity but lower specificity compared to the MoCA *z*-score, and lower sensitivity but higher specificity compared to the uncorrected MoCA score. This result is in line with previous findings from a simulation [33]. Whether to rely on a demographically adjusted score or on an uncorrected raw score may depend on the setting. For instance, when the MoCA is applied to identify cognitively healthy participants in clinical research, high sensitivity might be more important to avoid the inclusion of patients with false-negative test results. In contrast, if the aim is to include cognitively impaired patients in a clinical trial, high specificity should be favored over sensitivity to avoid including healthy individuals with false-positive results. Indeed, the erroneous inclusion of cognitively healthy individuals as patients may mask possible treatment effects in clinical trials [4]. When a general practitioner should decide whether to refer a patient to a specialized Memory Clinic based on cognitive screening, false-positive results should be minimized to reduce healthcare costs and discomfort for the individual. On the other hand, false-negative results may deprive a patient of the early implementation of therapeutic strategies. In this situation, we suggest relying on our new system with two separate cut-offs and an indecisive area.

### Limitations

Sensitivity, specificity, and the AUC give an indication of the quality of the test under observation by classifying the test performance with respect to a reference standard (i.e., an individual will be classified as a patient on the MoCA as well as according to a complete Memory Clinic diagnostic workup). However, these measures do not inform about the probability whether a tested individual has a specific disease [15, 34]. Predictive values―which are influenced by prevalence rates―reflect this information. In the current study, the MoCA had very high PPV across all patient groups and most MoCA scores (see Supplementary Fig. 2). However, the PPV will be lower in a setting with low prevalence of a specific disease (e.g., when screening for cognitive impairment at the general practitioner’s office). Likewise, in most MoCA studies reporting PPV and NPV, the prevalence of MCI was greater than in the general population [10]. Ideally, the diagnostic accuracy of a test should be evaluated in the same setting where it is clinically applied [35]. We did not have access to any data from first step screening processes (i.e., from a general practitioner’s office). Thus, our findings inform about how well the MoCA classifies individuals as healthy or cognitively impaired compared to a more extensive, multi-dimensional, diagnostic process, as performed in our Memory Clinic (described in the “Procedures” section). Additionally, we can provide the probability for a Memory Clinic patient to be affected by a mild or major NCD, when the MoCA performance is below the cut-off (PPV), as well as the probability that the patient is cognitively healthy, when the performance lies above the cut-off (NPV) [34]. This should be kept in mind, when applying our findings to other settings than a Memory Clinic. We refer to the excellent recent publication by Trevethan [34] for a better understanding on the informative value of sensitivity, specificity, PPV, and NPV.

There may be a selection bias in the NC and MC groups. The NC group was recruited from a Registry of Individuals Interested to Participate in Research, which may exhibit a greater motivation to perform well in the neuropsychological assessment. The MC group consisted of patients referred to the Memory Clinic from external medical professionals (e.g., general practitioners, hospitals) and may differ from individuals not seeking advice from a medical professional.

## Conclusion

In the present study, the diagnostic properties of the German MoCA were evaluated in an outpatient sample referred to a university-affiliated Memory Clinic. The originally proposed MoCA cut-off (25/26 points) had good sensitivity for mild and major NCD, but specificity was poor. As an alternative, a cut-off of 23/24 points on the MoCA improved specificity. However, the sensitivity to detect mild NCD was low using this cut-off. Thus, both cut-offs lead to a trade-off in either sensitivity (23/24 points) or specificity (25/26 points). In this context, we propose a new method to guide clinical decision-making by relying on two separate cut-offs combined with an indecisive area. Adding an indecisive area will increase both, sensitivity and specificity. Moreover, the presence of an indecisive area highlights the difficulties related to the early detection of cognitive impairment and mirrors the clinical reality quite accurately.

## Supplementary information

**Additional file 1: Supplementary Table 1.** Diagnoses in the patient sample. **Supplementary Figure 1**. ROC curves. **Supplementary Figure 2.** Positive and negative predictive values. Excel file for automatic calculation of demographically corrected z-score for the German version of the MocA. MoCA z-score calculation

## Data Availability

The datasets analyzed during the study are available from the corresponding author on reasonable request.

## Abbreviations

AD: Alzheimer’s disease
AUC: Area under the curve
CERAD-NAB: Consortium to Establish a Registry for Alzheimer’s Disease-Neuropsychological Assessment Battery
DSM-5: Diagnostic and Statistical Manual, 5th Edition
EKNZ: *Ethikkommission Nordwest- und Zentralschweiz* (i.e., regional research ethics board)
GDS: Geriatric Depression Scale
MCI: Mild cognitive impairment
MMSE: Mini Mental Status Examination
MoCA: Montreal Cognitive Assessment
NC: Normal controls
NCD: Neurocognitive disorder
NF: Normal findings
NPV: Negative predictive values
PPV: Positive predictive values
ROC: Receiver operating characteristic
