# Supplementary material for: Enhanced diagnostic accuracy for neurocognitive disorders: a revised cut-off approach for the Montreal Cognitive Assessment
Source: Alzheimers Res Ther. 2020 Apr 7;12:39. doi: 10.1186/s13195-020-00603-8 (PMC7140337; doi:10.1186/s13195-020-00603-8)
Supplement: Supplementary file 1 — Additional file 1: Supplementary Table 1. Diagnoses in the patient sample. Supplementary Figure 1. ROC curves. Supplementary Figure 2. Positive and negative predictive values. Excel file for automatic calculation of demographically corrected z-score for the German version of the MocA. MoCA z-score calculation [file 13195_2020_603_MOESM1_ESM.zip › Supplementary Table 1.docx]

**Supplementary Table 1.** Diagnoses in the patient sample.

| **Diagnoses** | **Mild NCD** | **Major NCD** | **Total** |
| --- | --- | --- | --- |
| Alzheimer’s clinical syndrome^1^ | 55 | 237 | 292 |
| Vascular cognitive impairment^2, 3^ | 15 | 12 | 27 |
| Lewy Body disease^4^ | 1 | 3 | 4 |
| Behavioral variant fronto-temporal dementia^5^ | 0 | 4 | 4 |
| Parkinson’s disease^6^ | 3 | 5 | 8 |
| Multiple system atrophy^7^ | 0 | 2 | 2 |
| Progressive supranuclear palsy^8^ | 2 | 0 | 2 |
| Primary progressive aphasia^9^ | 2 | 2 | 4 |
| Posterior cortical atrophy^10^ | 0 | 2 | 2 |
| Psychiatric disorder | 7 | 1 | 8 |
| Obstructive sleep apnea | 4 | 0 | 4 |
| Sleep disorder | 3 | 0 | 3 |
| Uncertain | 39 | 9 | 48 |
| Other | 28 | 11 | 39 |

1. Jack CR, Jr., Bennett DA, Blennow K et al. NIA-AA Research Framework: Toward a biological definition of Alzheimer's disease. Alzheimers Dement 2018;14:535-562.

2. Sachdev P, Kalaria R, O'Brien J et al. Diagnostic criteria for vascular cognitive disorders: a VASCOG statement. Alzheimer Dis Assoc Disord 2014;28:206-218.

3. Smith E. Vascular Cognitive Impairment. Continuum (Minneap Minn) 2016;22:490-509.

4. McKeith IG, Boeve BF, Dickson DW et al. Diagnosis and management of dementia with Lewy bodies. Fourth consensus report of the DLB Consortium 2017.

5. Rascovsky K, Hodges JR, Knopman D et al. Sensitivity of revised diagnostic criteria for the behavioural variant of frontotemporal dementia. Brain 2011;134:2456-2477.

6. Emre M, Aarsland D, Brown R et al. Clinical diagnostic criteria for dementia associated with Parkinson's disease. Mov Disord 2007;22:1689-1707; quiz 1837.

7. Palma JA, Norcliffe-Kaufmann L, Kaufmann H. Diagnosis of multiple system atrophy. Auton Neurosci 2018;211:15-25.

8. Hoglinger GU, Respondek G, Stamelou M et al. Clinical diagnosis of progressive supranuclear palsy: The movement disorder society criteria. Mov Disord 2017;32:853-864.

9. Gorno-Tempini ML, Hillis AE, Weintraub S et al. Classification of primary progressive aphasia and its variants. Neurology 2011;76:1006-1014.

10. Crutch SJ, Schott JM, Rabinovici GD et al. Consensus classification of posterior cortical atrophy. Alzheimers Dement 2017;13:870-884.

**Supplementary Figure 1. ROC curves.**

ROC curves for the MoCA (z-score: solid line, corrected score: dashed line) and the MMSE (dotted line) for the classification of Mild+Major NCD (Figure 1A) and Mild NCD (Figure 1B).

**Supplementary Figure 2.** Positive and negative predictive values.

In Supplementary Figure 1A, the positive predictive values (PPV) and negative predictive values (NPV) are plotted for Mild NCD vs. NF and highlighted for the proposed cut-offs of 23/24 points and 26/27 points. In Supplementary Figure 1B, PPV and NPV are illustrated for Mild+Major NCD vs. NF. In all patient groups, PPV decrease and NPV increase with higher MoCA threshold scores. Again, using two separate cut-offs enhances both, PPV and NPV.
